# Predictive role of geriatric nutritional risk index for postoperative complications in operated esophageal cancer patients: a meta-analysis

**DOI:** 10.1186/s13019-025-03703-4

**Published:** 2025-11-26

**Authors:** Long Tian, Yijie Bu, Yao Wang, Yan Wang, Guowei Che

**Affiliations:** 1 Department of Thoracic Surgery/Lung Cancer Center, West China Hospital, Lung Cancer Center, Sichuan University, Chengdu, 610041 P.R. China; 2 Department of Thoracic Surgery, West China Hospital, Sichuan University, Chengdu, 610041 P.R. China

**Keywords:** Geriatric nutritional risk index, Esophageal cancer, Postoperative complication, Meta-analysis

## Abstract

**Purpose:**

To determine the association between preoperative geriatric nutritional risk index (GNRI) and postoperative complications in surgical esophageal cancer patients.

**Methods:**

PubMed, Web of Science EMBASE and CNKI databases were searched from inception up to December 16, 2024. Primary outcome was the overall complication. Secondary outcomes included the postoperative pulmonary complication (PPC), anastomotic leakage, chylothorax, vocal code paresis, pneumonia, arrhythmia, incision infection, gastrointestinal complication, respiratory failure and heart failure. Odds ratios (ORs) and 95% confidence intervals (CIs) were combined.

**Results:**

Eight eligible studies were included with 1,689 cases. Pooled results demonstrated that lower GNRI was significantly related to increased risk of overall complication (OR = 2.56, 95% CI: 1.85–3.55, *P*<0.001) and PPC (OR = 2.06, 95% CI: 1.71–2.48, *P*<0.001). Besides, GNRI was associated with the risk of anastomotic leakage (OR = 1.83, *P* = 0.007), pneumonia (OR = 3.87, *P*<0.001) and respiratory failure (OR = 9.71, *P* = 0.003).

**Conclusion:**

Based on available evidence, GNRI could serve as a prognostic indicator and a lower GNRI predicted increased incidence of postoperative complications among surgical esophageal cancer patients. However, due to the limitations of this meta-analysis like the small sample size, more high-quality studies are still needed to verify above findings.

**Trial registration:**

This meta-analysis was registered in INSPLAY platform (INPLASY202590012; DOI: 10.37766/inplasy2025.9.0012).

**Supplementary Information:**

The online version contains supplementary material available at 10.1186/s13019-025-03703-4.

## Introduction

Esophageal cancer is a significant global health burden, ranking as one of the leading causes of cancer-related mortality worldwide [1]. The disease is particularly prevalent in certain regions, such as East Asia, where lifestyle and dietary factors contribute to its high incidence [2]. Surgical resection remains the cornerstone of curative treatment for resectable esophageal cancer, offering the best chance for long-term survival. However, esophagectomy is a complex and high-risk procedure often associated with substantial postoperative complications, including anastomotic leakage, respiratory infections, and cardiovascular events [3, 4]. These complications can significantly affect patient outcomes, prolong hospital stays, and increase mortality risk [5]. Predicting postoperative complications remains a clinical challenge, as existing predictive models often lack sufficient accuracy, generalizability, or applicability to diverse patient populations.

The Geriatric Nutritional Risk Index (GNRI), based on the serum albumin level, usual weight and ideal weight, is a valuable tool for assessing nutritional status and has shown significant prognostic value in cancer patients [6]. In long-term oncological outcomes, GNRI has been reported to be associated with survival rates, as poor nutritional status often correlates with diminished immune function, increased tumor progression, and reduced tolerance to treatments such as chemotherapy or radiotherapy [6, 7]. In the context of surgical oncology, GNRI has demonstrated utility in predicting short-term postoperative outcomes [8, 9]. For various malignancies, including gastrointestinal and thoracic cancers, low GNRI scores have been associated with a higher risk of postoperative complications such as infections, delayed wound healing, and increased morbidity [8, 9]. This highlights the GNRI’s potential as a practical and easily implementable marker to guide preoperative risk stratification and optimize perioperative care in cancer patients undergoing surgery. Esophageal cancer patients are generally elderly and prone to nutritional problems, both of which are closely associated with postoperative short-term risks. Therefore, the GNRI may have significant predictive value for short-term postoperative outcomes in patients undergoing esophageal cancer surgery, making it worthy of further clarification.

Therefore, this meta-analysis aimed to further determine the predictive role of preoperative GNRI for the risk of postoperative complications among operated esophageal cancer patients for the first time.

## Materials and methods

The current meta-analysis was performed according to the Preferred Reporting Items for Systematic Reviews and Meta-Analyses 2020 [10]. This meta-analysis was registered in INSPLAY platform (INPLASY202590012; DOI: 10.37766/inplasy2025.9.0012).

### Literature search

We searched the PubMed, Web of Science, EMBASE and CNKI databases from inception up to December 16, 2024 with following terms: geriatric nutritional risk index, GNRI, esophageal, esophagus, tumor, cancer, carcinoma and neoplasm. Both Medical Subject Headings (MeSH) and free-text terms were applied. The following search strategy was used in PubMed and adapted for other databases: (“geriatric nutritional risk index” OR GNRI) AND (“esophageal” OR “esophagus”) AND (“tumor” OR “cancer” OR “carcinoma” OR “neoplasm”).

## Inclusion criteia

Studies met following criteria were included: (1) patients received the esophageal operation due to the esophageal cancer; (2) GNRI was calculated before the surgery according to the formula: 1.489 × albumin (g/dl) + 41.7 × usual weight/ideal weight [11]; (3) patients were divided into different groups according to the GNRI and the incidence of complications between groups were compared; (4) odds ratios (ORs) with 95% confidence intervals (CIs) were reported or enough data were provided to calculate them; (5) full texts were available and articles were published in English or Chinese.

## Exclusion criteria

Studies met following criteria were excluded: (1) 1) case reports, reviews, animal trials, letters, meeting abstracts or editorials; (2) duplicated or overlapped data.

## Data extraction

Following data were collected from included studies: the name of first author, publication year, country, sample size, age, tumor stage, pathological type, history of neoadjuvant therapy, cutoff value of GNRI, endpoint, OR and 95% CI.

Primary outcome was the overall complication, which was defined as the incidence of any postoperative complication reported in each included study, regardless of type or severity. All complications documented by the original authors (e.g., pulmonary, cardiac, infectious, renal, bleeding, or other procedure-related events) were counted toward the overall complication rate. Secondary outcomes included the postoperative pulmonary complication (PPC), anastomotic leakage, chylothorax, vocal code paresis, pneumonia, arrhythmia, incision infection, gastrointestinal complication, respiratory failure and heart failure.

## Methodological quality assessment

In this meta-analysis, the quality of included studies were evaluated by the Newcastle-Ottawa Scale (NOS) score tool and studies with a NOS score>5 were defined as high-quality studies [12].

Two authors independently performed the literature search, selection, data extraction, and methodological quality assessment. Any discrepancies encountered during these processes were initially documented individually, followed by a structured team discussion involving a third senior investigator. During these discussions, authors revisited the original studies to clarify uncertainties, reassessed the extracted data points or quality criteria, and ultimately reached consensus through deliberation. This approach ensured accuracy, consistency, and objectivity in data extraction and quality evaluation.

### Statistical analysis

All statistical analyses were conducted using STATA (version 15.0) software. Heterogeneity between included studies was assessed by I^2^ statistics. If significant heterogeneity was detected (I^2^ >50%), the random-effects model was applied; otherwise, the fixed-effects model was applied. ORs and 95% CIs were combined to evaluate the predictive role of postoperative complications among surgical esophageal cancer patients. Sensitivity analysis by excluding each included studies at one time was conducted to detect the sources of heterogeneity and assess the stability of the overall results. Meanwhile, Begg’s funnel plot and Egger’s test were conducted to detect publication bias. If significant publication bias was detected as *P* < 0.05 [13, 14], the trim-and-fill method was applied to identify potentially unpublished studies [15].

## Results

### Literature search and selection

**Fig. 1:**
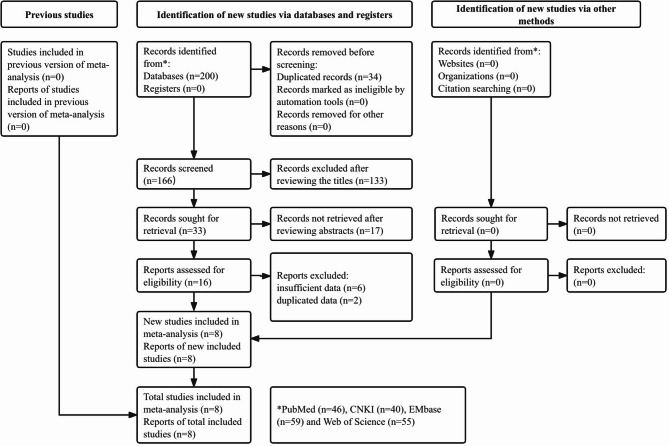
Flow diagram of this meta-analysis

Two hundred records were identified from databases and 34 duplicated records were removed. After reviewing the titles, abstracts and full texts, 133, 17 and 8 publications were excluded. Eventually, eight eligible studies were included [16–23]. The specific process was presented in Fig. 1.

### Basic characteristics of included studies

Among eight studies, 1,689 patients were included. All studies were from China (6/8) and Japan (2/8) with the sample size ranged from 90 to 373. Most studies focused on squamous cell carcinoma (SCC) and elderly patients. The cutoff values of GNRI ranged from 90 to 102.6 and all studies were high-quality studies. The study by Wang et al. [19] involved two groups of patients. Therefore, we regarded them as two independent studies during the analyses. Detailed data were shown in Table 1. All studies were with high-quality and the information about the NOS scores of each included studies was presented in Supplementary Table 1.

**Table 1:** Basic characteristics of included studies

Author	Year	Country	Sample size	Age	Tumor stage	Pathological type	Neoadjuvant therapy	Cutoff value of GNRI	Endpoint	NOS
Yamana [16]	2015	Japan	122	63.9 ± 9.1	I-IV	NR	Mixed	90	PPC, AL	6
Hong [17]	2019	China	173	61.08 ± 9.255	I-III	SCC	NR	92	Complication, PPC, AL, GIC, II, chylothorax, CVC, LKD	7
Kubo [18]	2019	Japan	240	63.4 ± 7.8	II-III	SCC	CT/CRT	92	PPC, AL, II, chylothorax, VCP	7
Wang [19]	2021	China	192	65.1 ± 7.2	I-IVA	SCC	Mixed	92	Complication, PPC, AL, GIC, pneumonia, arrhythmia, RF, chylothorax, VCP, HF	7
Wang [19]	2021	China	155	62.5 ± 8.6	I-IVA	SCC	Mixed	92	Complication, PPC, AL, GIC, pneumonia, arrhythmia, RF, chylothorax, VCP, HF	7
Fang [20]	2022	China	373	NR	NR	SCC	CT/CRT	98.7	PPC, AL	7
Zhang [21]	2022	China	90	NR	NR	SCC	None	98	Complication	6
Liang [22]	2023	China	120	62.82(mean)	NR	NR	NR	NR	Complication, AL, pneumonia, arrhythmia, chylothorax	7
Feng [23]	2024	China	224	63.1 ± 6.7	II-IVA	SCC	IT	102.6	Complication, PPC, AL, VCP	7

PPC: postoperative pulmonary complication; AL: anastomotic leakage; GIC: gastrointestinal complication; II: incision infection; CVC: cardiovascular complication; LKD: liver and kidney dysfunction; VCP: vocal code paresis; RF: respiratory failure; HF: heart failure; CT: chemotherapy; CRT: chemoradiotherapy; IT: immunotherapy; NR: nor reported; NOS: Newcastle-Ottawa Scale; GRNI: geriatric nutritional risk index

### Association of GRNI with primary outcome in surgical esophageal cancer

**Fig. 2:**
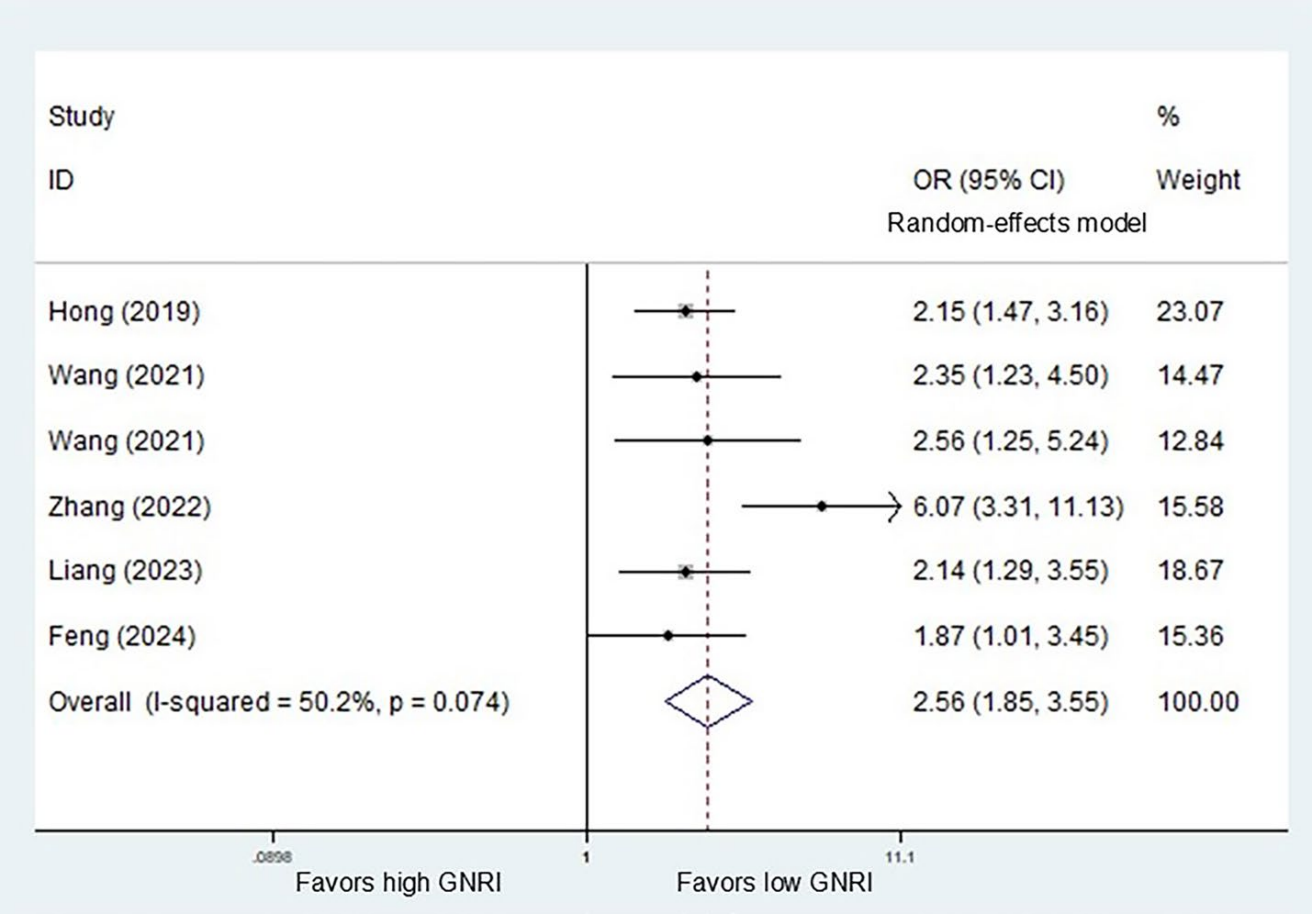
Association of preoperative geriatric nutritional risk index with postoperative overall complications among surgical esophageal cancer patients

Five studies explored the relationship between GNRI and overall complication. Pooled results demonstrated that a lower preoperative GNRI predicted increased risk of overall complications (OR = 2.56, *P*<0.001) (Fig. 2).

### Association of GRNI with secondary outcomes in surgical esophageal cancer

**Fig. 3:**
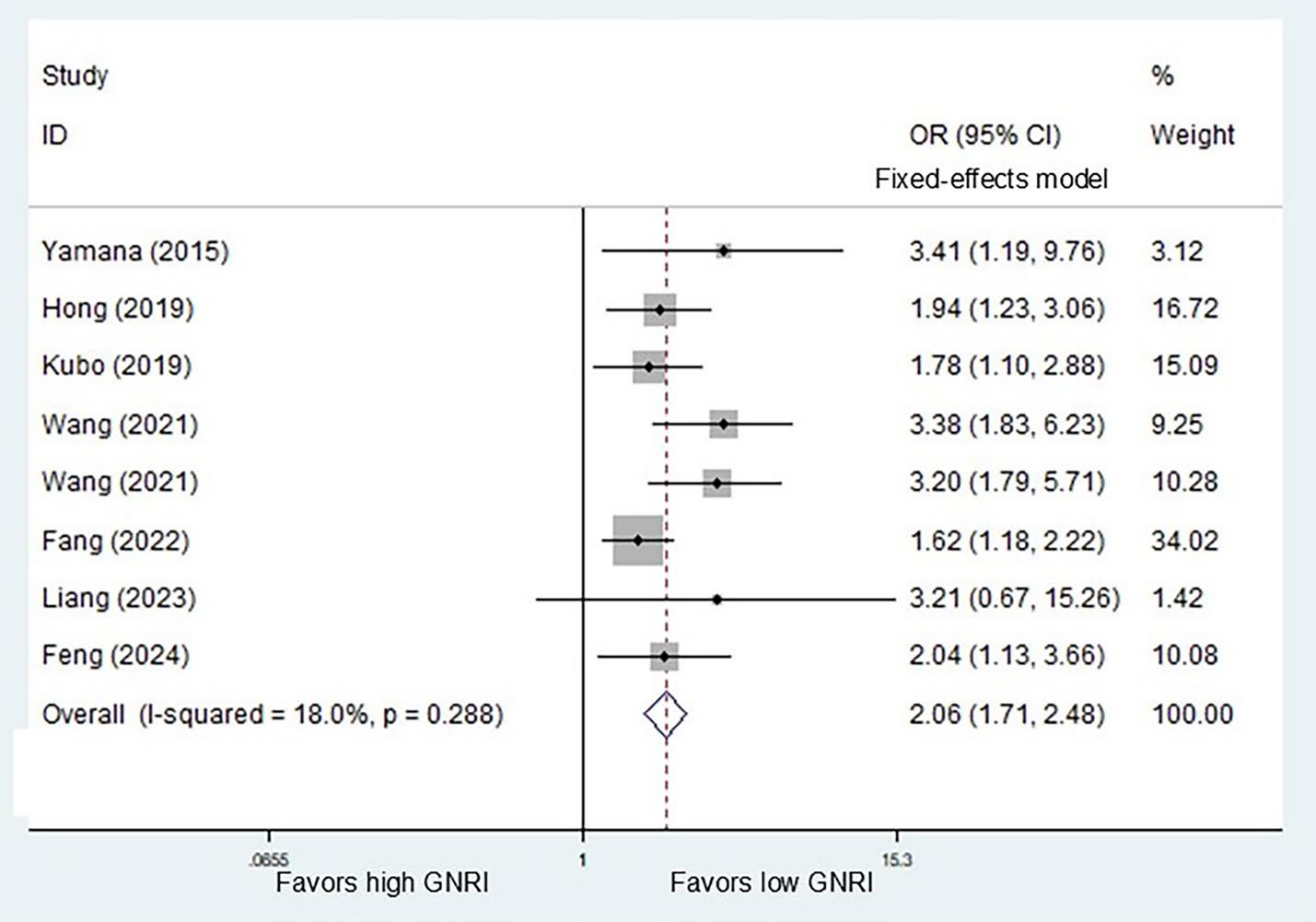
Association of preoperative geriatric nutritional risk index with postoperative pulmonary complications among surgical esophageal cancer patients

According to the pooled results, preoperative GNRI was also associated with PPCs (OR = 2.06, *P*<0.001) (Fig. 3), anastomotic leakage (OR = 1.83, *P* = 0.007) (Supplementary Fig. 1A), pneumonia (OR = 3.87, *P*<0.001) (Supplementary Fig. 1B) and respiratory failure (OR = 9.71, *P* = 0.003). However, no significantly relationship between GNRI and chylothorax (OR = 1.79, *P* = 0.118) (Supplementary Fig. 1C), vocal code paresis (OR = 0.99, *P* = 0.947) (Supplementary Fig. 1D), arrhythmia (OR = 1.58, *P* = 0.082) (Supplementary Fig. 1E), incision infection (OR = 0.81, *P* = 0.589) (Supplementary Fig. 1F), gastrointestinal complication (OR = 2.00, *P* = 0.080) (Supplementary Fig. 1G)or heart failure (OR = 3.70, *P* = 0.366) were observed. (Table 2)

**Table 2:** Results of meta-analysis

Items	No. of studies	OR	95% CI	*P* value	I^2^ (%)	*P* value
Primary outcomes						
Overall complication	5	2.56	1.85–3.55	<0.001	50.2	0.074
Secondary outcomes						
Postoperative pulmonary complication	7	2.06	1.71–2.48	<0.001	18.0	0.288
Anastomotic leakage	6	1.83	1.18–2.83	0.007	47.3	0.077
Chylothorax	4	1.79	0.86–3.69	0.118	0.0	0.870
Vocal code paresis	3	0.99	0.67–1.45	0.947	10.9	0.338
Pneumonia	2	3.87	1.88–7.97	<0.001	55.3	0.107
Arrhythmia	2	1.58	0.94–2.63	0.082	38.0	0.200
Incision infection	2	0.81	0.38–1.73	0.589	0.0	0.489
Gastrointestinal complication	2	2.00	0.92–4.36	0.080	43.3	0.172
Respiratory failure	1	9.71	2.13–44.25	0.003	0.0	0.975
Heart failure	1	3.70	0.22–62.84	0.366	71.3	0.062

OR: odds ratio; CI: confidence interval; PPC: postoperative pulmonary complication

### Sensitivity analysis

**Fig. 4:**
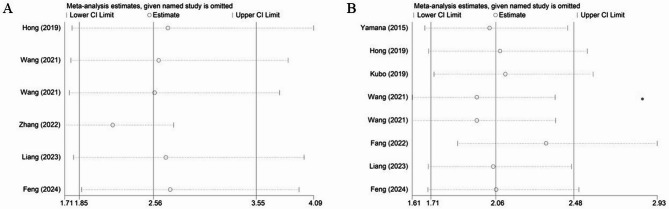
Sensitivity analysis for the association of preoperative geriatric nutritional risk index with overall complication (**A**) and postoperative pulmonary complications (**B**) among surgical esophageal cancer patients

Sensitivity analysis by excluding each included studies at one time for the overall complication (Fig. 4A) and PPC (Fig. 4B) was performed, which manifested that our results were stable and none of included studies caused an obvious impact on the overall findings.

### Publication bias

**Fig. 5:**
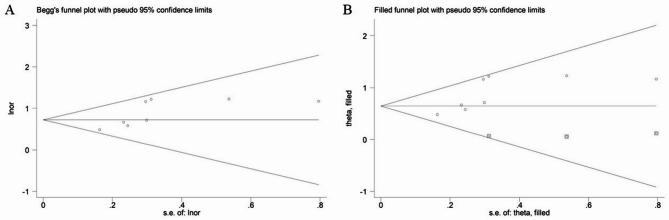
Begg’s (**A**) and filled (**B**) funnel plots for the association of preoperative geriatric nutritional risk index with postoperative pulmonary complications among surgical esophageal cancer patients

According to the Begg’s funnel plot (Fig. 5A) and Egger’s test (*P* = 0.043), significant publication bias was observed. Therefore, the trim-and-fill method was used and three potentially unpublished studies were found (Fig. 5B). However, these three studies did not affect the overall conclusion (filled OR = 1.90, 95% CI: 1.60–2.26, *P*<0.001).

## Discussion

This meta-analysis demonstrated that preoperative GNRI was significantly associated with postoperative complications among operated esophageal cancer patients. In detail, a lower GNRI predicted increased incidence of overall complication, PPC, anastomotic leakage, pneumonia and respiratory failure. Therefore, GNRI could serve as a prognostic indicator for the risk evaluation and clinical management for esophageal cancer patients receiving the surgery.

Several nutritional and inflammation-based indices, such as the modified GNRI (mGNRI), Glasgow Prognostic Score (GPS), modified GPS (mGPS), and the C-reactive protein-to-albumin ratio (CAR), have also been proposed as prognostic tools in oncology. However, these indices often require inflammatory markers such as C-reactive protein, which may not be routinely available in preoperative assessments across all institutions and can be influenced by transient conditions unrelated to nutritional status. In contrast, the GNRI is calculated solely from serum albumin and body weight parameters, making it simple, objective, and widely applicable in surgical settings. Moreover, GNRI was originally designed to evaluate nutritional risk in elderly patients, a population particularly relevant to esophageal cancer, and has shown consistent prognostic value across various malignancies. These characteristics make GNRI a practical and clinically feasible index for assessing perioperative risk in esophageal cancer patients.

Our findings suggest that GNRI has practical clinical value in the perioperative management of esophageal cancer patients. Preoperative GNRI screening is simple, inexpensive, and based on routinely available parameters, making it a feasible tool for early identification of patients at high risk of postoperative complications. Recognizing these high-risk individuals allows clinicians to implement targeted nutritional optimization, intensified perioperative monitoring, and preventive interventions for pulmonary complications or anastomotic leakage. Therefore, incorporating GNRI into preoperative assessment may help improve surgical outcomes and reduce postoperative morbidity in esophageal cancer patients.

GNRI predicts the risk of postoperative complications in esophageal cancer primarily by comprehensively reflecting the patient’s overall nutritional status and health reserves. Malnutrition is a common issue in esophageal cancer patients, especially preoperatively, due to dysphagia and cancer-related cachexia, which lead to insufficient nutritional intake [24]. Low GNRI scores typically indicate inadequate protein and energy intake, closely associated with weakened immune function. Impaired immunity increases the risk of infections, delays wound healing, and reduces postoperative recovery capacity, thereby leading to complications [25]. The GNRI calculation formula is based on serum albumin levels and body mass index (BMI). Albumin is a sensitive indicator of systemic inflammation and nutritional status. Low albumin levels are significantly associated with poor wound healing, anastomotic leakage, and infections after surgery [15, 26]. GNRI also incorporates BMI as an indicator of physical reserves. Patients with low BMI often lack adequate fat and muscle mass, and the metabolic burden of the postoperative stress state may exacerbate these deficiencies, increasing the risk of complications [27]. Malnutrition is often accompanied by a systemic inflammatory state, which not only impairs tissue repair but also promotes the occurrence of complications, such as postoperative pulmonary infections and multiple organ dysfunction [28].

It should be noted that serum albumin, a core component of the GNRI, is not a specific biomarker of nutritional status. Albumin levels may be affected by inflammation, liver or kidney function, and hydration status in addition to nutrition. Therefore, while GNRI provides a simple and clinically feasible assessment, its predictive accuracy may be partially limited by the inherent shortcomings of albumin as a nutritional biomarker.

There are some limitations which should be noted. First of all, the number of included studies and overall sample size is relatively small, which might cause some bias. Second, all included studies were conducted in Asian populations (China or Japan), where the incidence of adenocarcinoma is relatively low. This histological selection bias may limit the generalizability of our findings to non-Asian populations. Meanwhile, this might also be the cause for the publication bias found. Third, we are unable to conduct more subgroup analysis based on some important factors such as the age, comorbidities, tumor stage, surgical procedure and neoadjuvant therapy due to the lack of original data and meta-regression analysis, which also limited the investigation of potential sources of heterogeneity. Four, in this type of study, it is unable to further determine the optimal cutoff value of GNRI for the prediction of postoperative complications. Five, our study could not evaluate the relationship between GNRI and outcomes such as length of hospital stay, readmission, or healthcare costs.

## Conclusion

According to our findings, GNRI could serve as a prognostic indicator, with a lower GNRI predicting an increased incidence of postoperative complications among surgical esophageal cancer patients. However, additional prospective studies are required to validate these findings due to the limitations inherent in this meta-analysis.

## Supplementary Information

Supplementary Material 1

Supplementary Material 2

## Data Availability

All data generated or analyzed during this study are included in this published article.
