# Supplementary material for: Predictive role of geriatric nutritional risk index for postoperative complications in operated esophageal cancer patients: a meta-analysis
Source: J Cardiothorac Surg. 2025 Nov 26;20:440. doi: 10.1186/s13019-025-03703-4 (PMC12659037; doi:10.1186/s13019-025-03703-4)

Supplementary figure 1A. Association of geriatric nutritional risk index with the incidence of anastomotic leakage in surgical esophageal cancer patients.


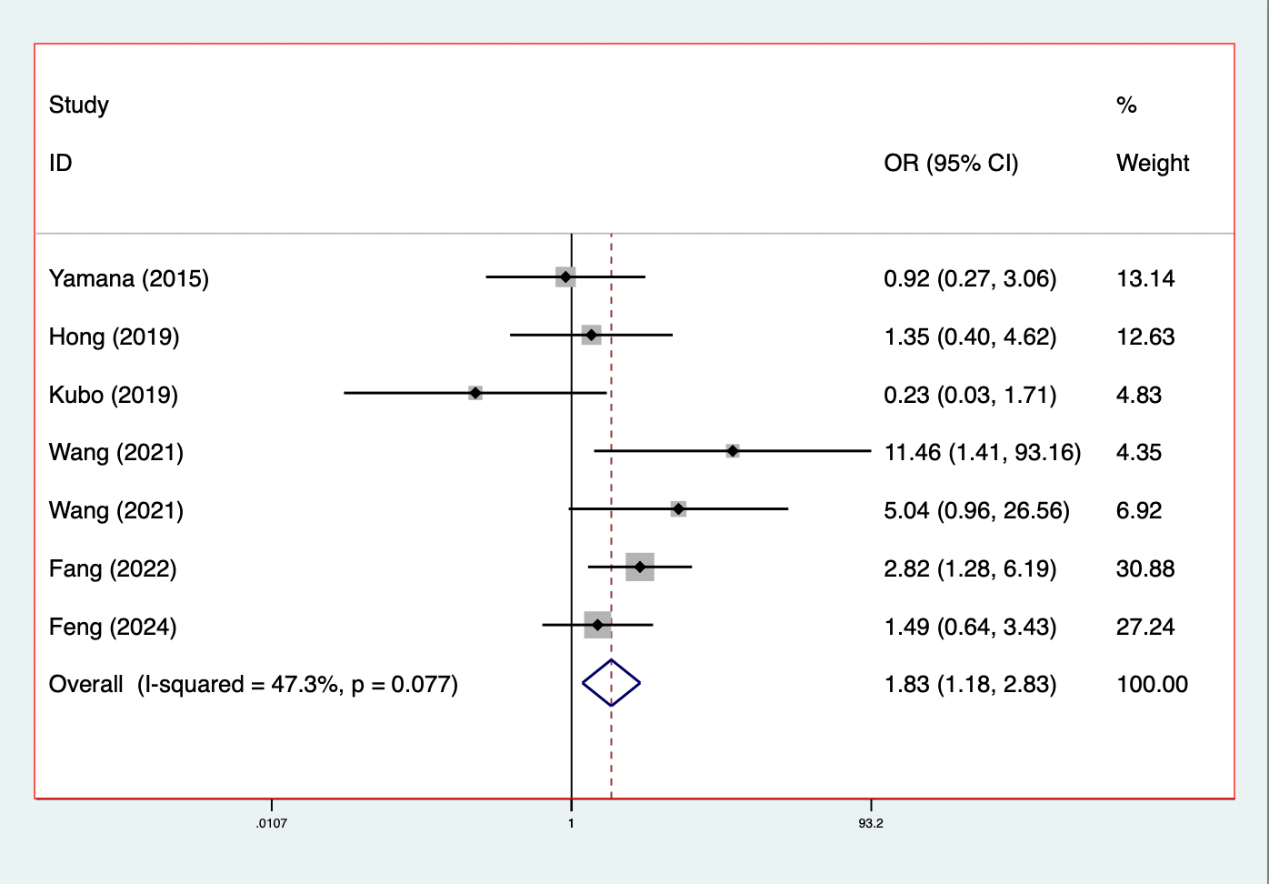


Supplementary figure 1B. Association of geriatric nutritional risk index with the incidence of pneumonia in surgical esophageal cancer patients.


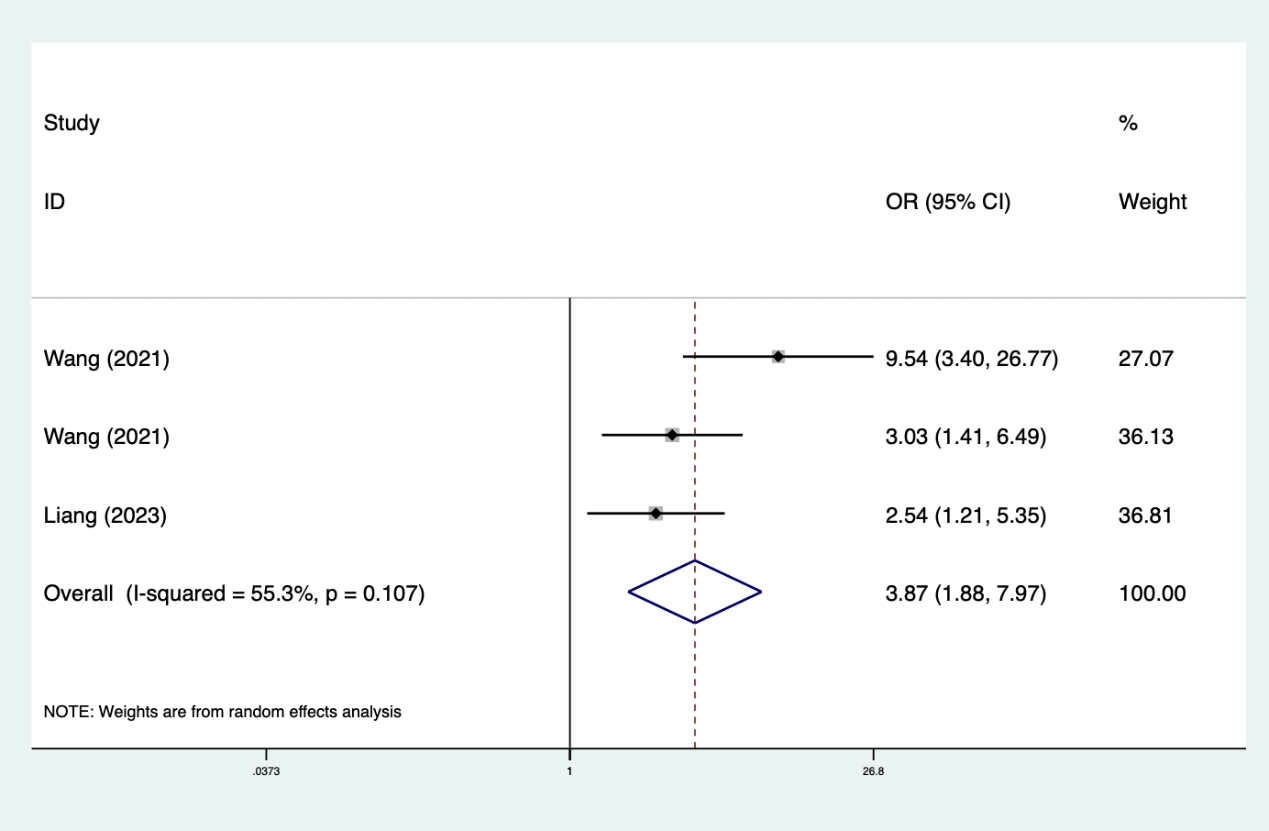


Supplementary figure 1C. Association of geriatric nutritional risk index with the incidence of chylothorax in surgical esophageal cancer patients.


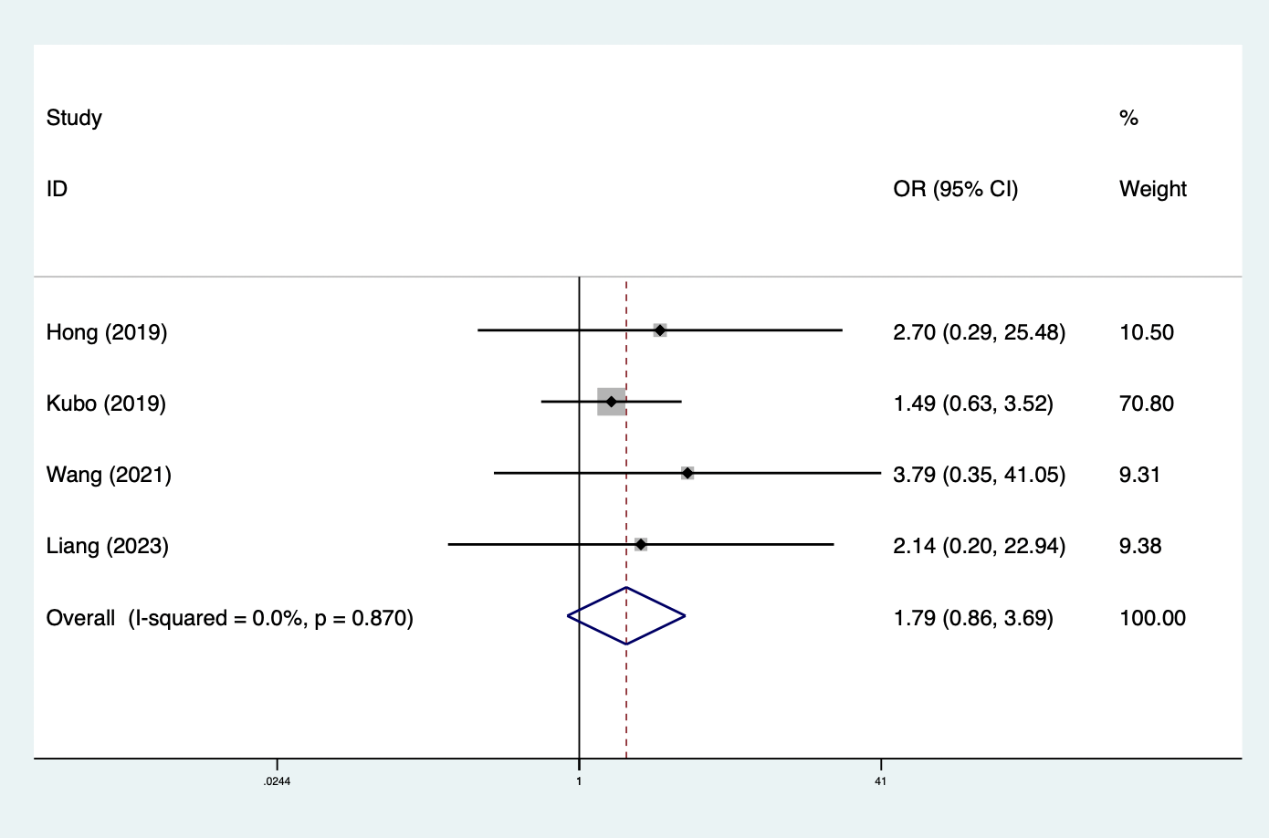


Supplementary figure 1D. Association of geriatric nutritional risk index with the incidence of vocal code paresis in surgical esophageal cancer patients.


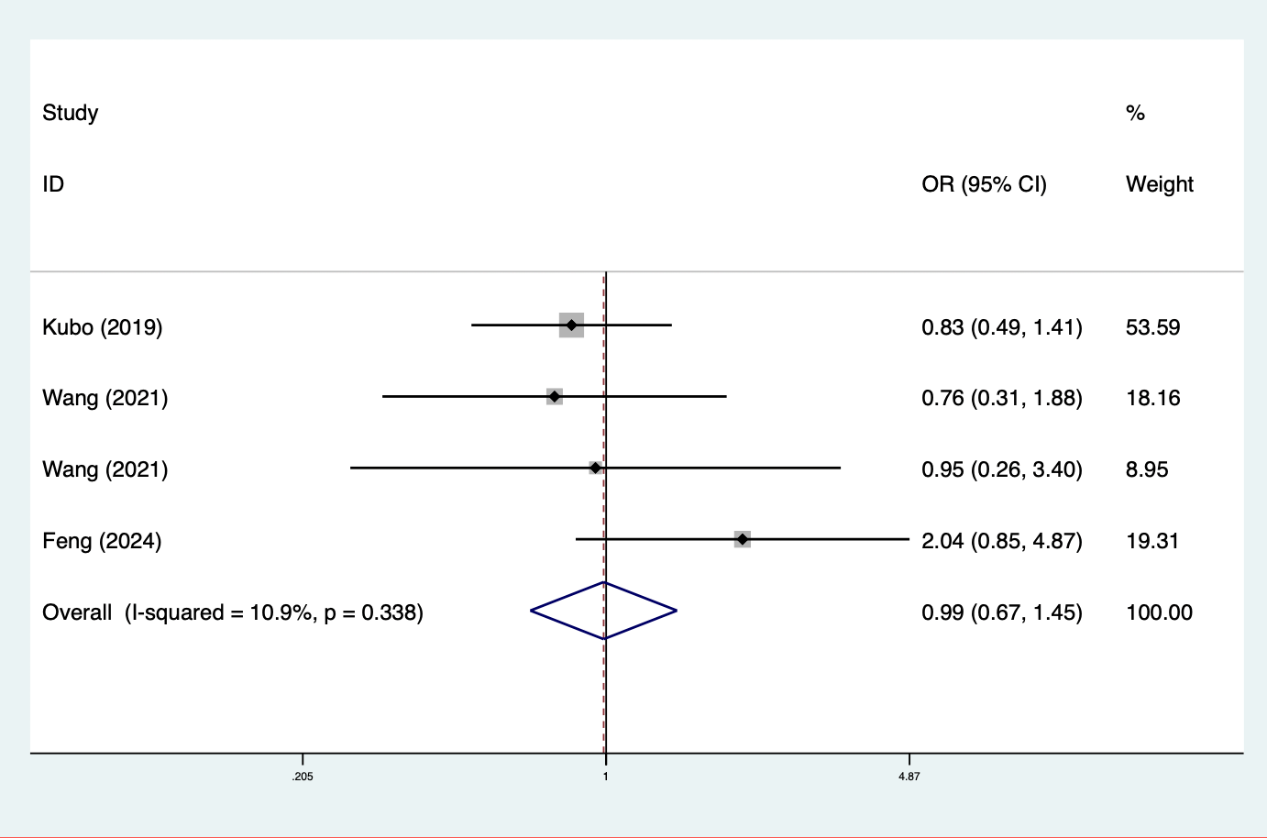


Supplementary figure 1E. Association of geriatric nutritional risk index with the incidence of arrhythmia in surgical esophageal cancer patients.


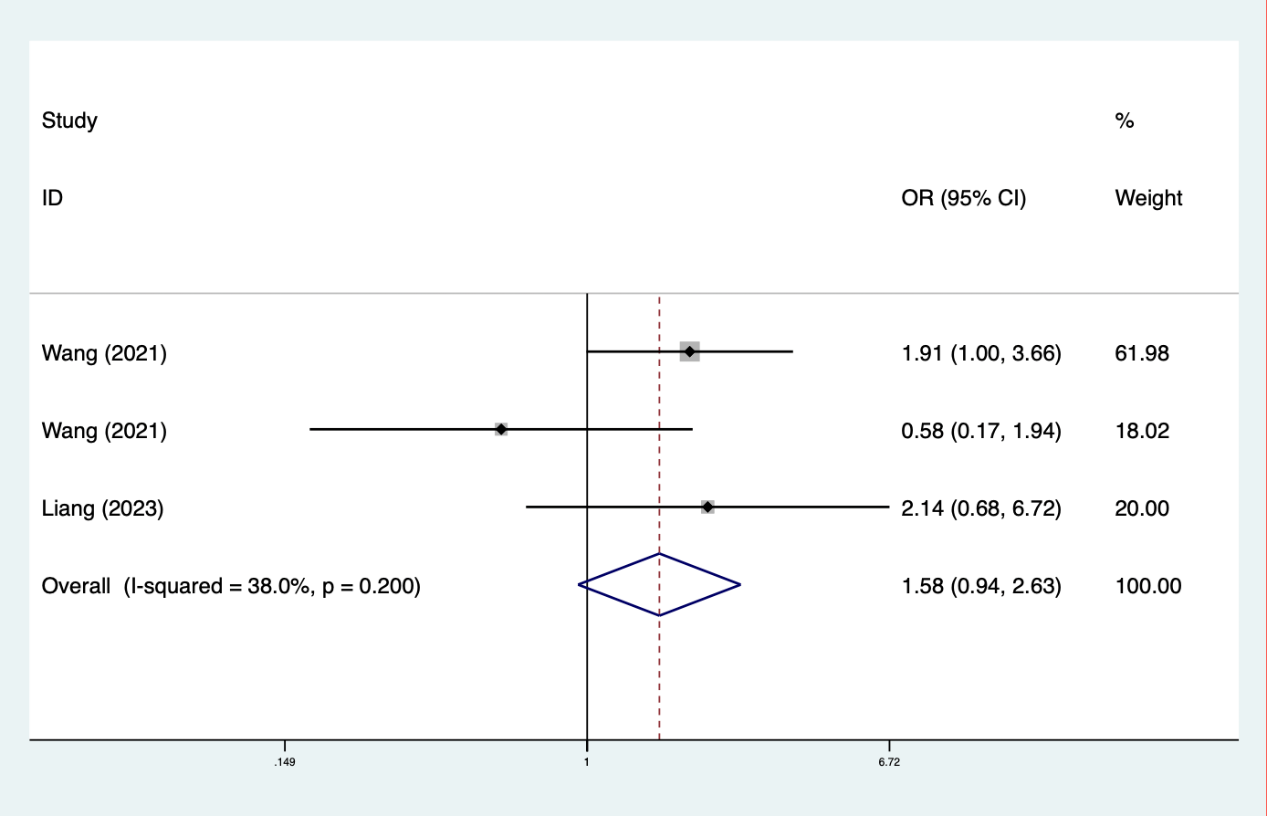


Supplementary figure 1F. Association of geriatric nutritional risk index with the incidence of incision infection in surgical esophageal cancer patients.


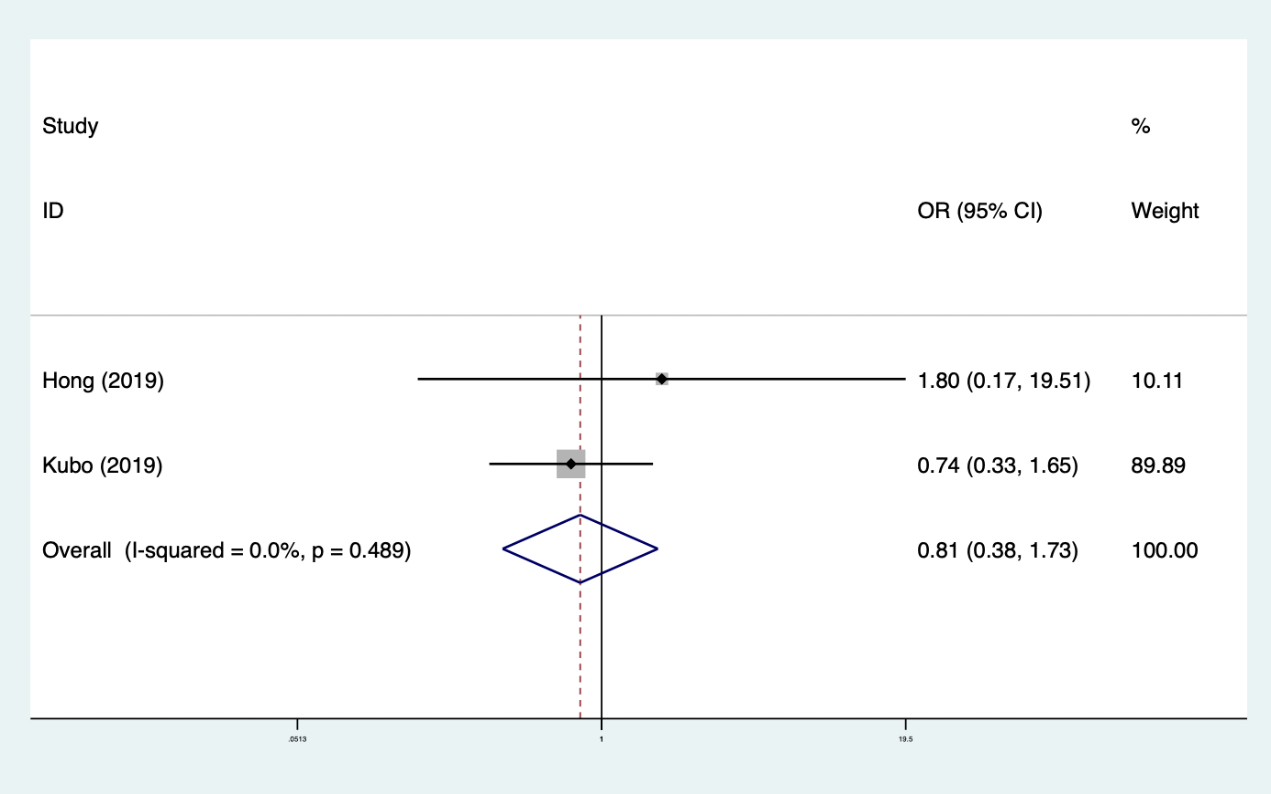


Supplementary figure 1G. Association of geriatric nutritional risk index with the incidence of gastrointestinal complication in surgical esophageal cancer patients.


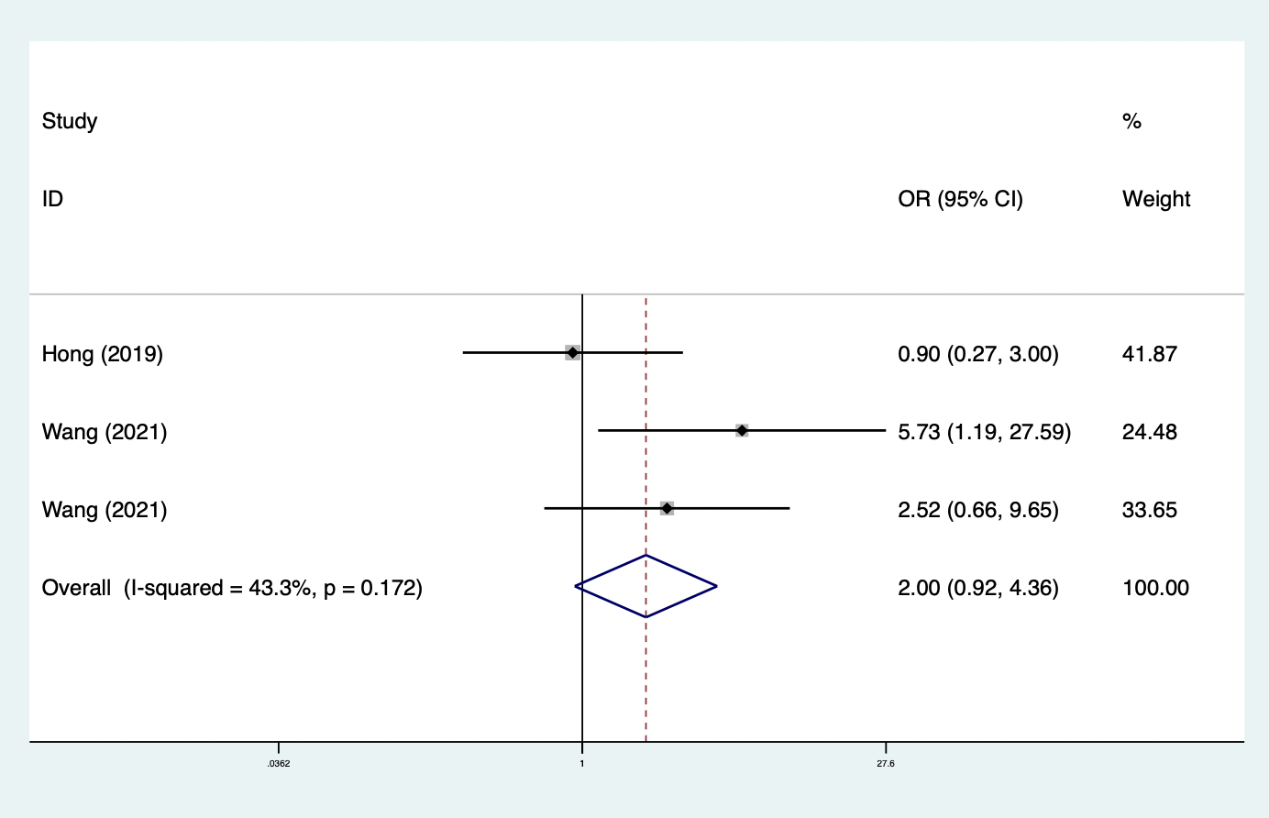

Supplement: Supplementary file 1 — Supplementary Material 1 [file 13019_2025_3703_MOESM1_ESM.docx]
