# Supplementary material for: Predictive role of geriatric nutritional risk index for postoperative complications in operated esophageal cancer patients: a meta-analysis
Source: J Cardiothorac Surg. 2025 Nov 26;20:440. doi: 10.1186/s13019-025-03703-4 (PMC12659037; doi:10.1186/s13019-025-03703-4)
Supplement: Supplementary file 2 — Supplementary Material 2 [file 13019_2025_3703_MOESM2_ESM.docx]

Supplementary table 1. Detailed results of NOS score.

| Author | Year | Selection | | | | Comparability | Outcome measurement | | | Score |
| --- | --- | --- | --- | --- | --- | --- | --- | --- | --- | --- |
|  |  | Representativeness of the exposed cohort | Selection of the non-exposed cohort | Ascertainment of exposure | Outcome of interest | Comparability of cohorts* | Assessment of outcome | Time of follow-up | Adequacy of follow-up |  |
| Yamana | 2015 | 1 | 0 | 1 | 1 | 0 | 1 | 1 | 1 | 6 |
| Hong | 2019 | 1 | 1 | 1 | 1 | 0 | 1 | 1 | 1 | 7 |
| Kubo | 2019 | 1 | 1 | 1 | 1 | 0 | 1 | 1 | 1 | 7 |
| Wang | 2021 | 1 | 1 | 1 | 1 | 0 | 1 | 1 | 1 | 7 |
| Wang | 2021 | 1 | 1 | 1 | 1 | 0 | 1 | 1 | 1 | 7 |
| Fang | 2022 | 1 | 0 | 1 | 1 | 0 | 1 | 1 | 1 | 6 |
| Zhang | 2022 | 1 | 1 | 1 | 1 | 0 | 1 | 1 | 1 | 7 |
| Liang | 2023 | 1 | 1 | 1 | 1 | 0 | 1 | 1 | 1 | 7 |

NOS: Newcastle-Ottawa Scale. * A maximum of two stages could be given for the Comparability.
